# Mitochondrial Functional State Impacts Spontaneous Neocortical Activity and Resting State fMRI

**DOI:** 10.1371/journal.pone.0063317

**Published:** 2013-05-01

**Authors:** Basavaraju G. Sanganahalli, Peter Herman, Fahmeed Hyder, Sridhar S. Kannurpatti

**Affiliations:** 1 Department of Diagnostic Radiology, Yale University School of Medicine, New Haven, Connecticut, United States of America; 2 Department of Biomedical Engineering, Yale University School of Medicine, New Haven, Connecticut, United States of America; 3 Department of Magnetic Resonance Research Center, Yale University School of Medicine, New Haven, Connecticut, United States of America; 4 Core Center for Quantitative Neuroscience with Magnetic Resonance, Yale University School of Medicine, New Haven, Connecticut, United States of America; 5 Department of Radiology, UMDNJ-New Jersey Medical School, Newark, New Jersey, United States of America; UPR 3212 CNRS -Université de Strasbourg, France

## Abstract

Mitochondrial Ca^2+^ uptake, central to neural metabolism and function, is diminished in aging whereas enhanced after acute/sub-acute traumatic brain injury. To develop relevant translational models for these neuropathologies, we determined the impact of perturbed mitochondrial Ca^2+^ uptake capacities on intrinsic brain activity using clinically relevant markers. From a multi-compartment estimate of probable baseline Ca^2+^ ranges in the brain, we hypothesized that reduced or enhanced mitochondrial Ca^2+^ uptake capacity would decrease or increase spontaneous neuronal activity respectively. As resting state fMRI-BOLD fluctuations and stimulus-evoked BOLD responses have similar physiological origins [1] and stimulus-evoked neuronal and hemodynamic responses are modulated by mitochondrial Ca^2+^ uptake capacity [2], [3] respectively, we tested our hypothesis by measuring hemodynamic fluctuations and spontaneous neuronal activities during normal and altered mitochondrial functional states. Mitochondrial Ca^2+^ uptake capacity was perturbed by pharmacologically inhibiting or enhancing the mitochondrial Ca^2+^ uniporter (mCU) activity. Neuronal electrical activity and cerebral blood flow (CBF) fluctuations were measured simultaneously and integrated with fMRI-BOLD fluctuations at 11.7T. mCU inhibition reduced spontaneous neuronal activity and the resting state functional connectivity (RSFC), whereas mCU enhancement increased spontaneous neuronal activity but reduced RSFC. We conclude that increased or decreased mitochondrial Ca^2+^ uptake capacities lead to diminished resting state modes of brain functional connectivity.

## Introduction

**Figure 1:**
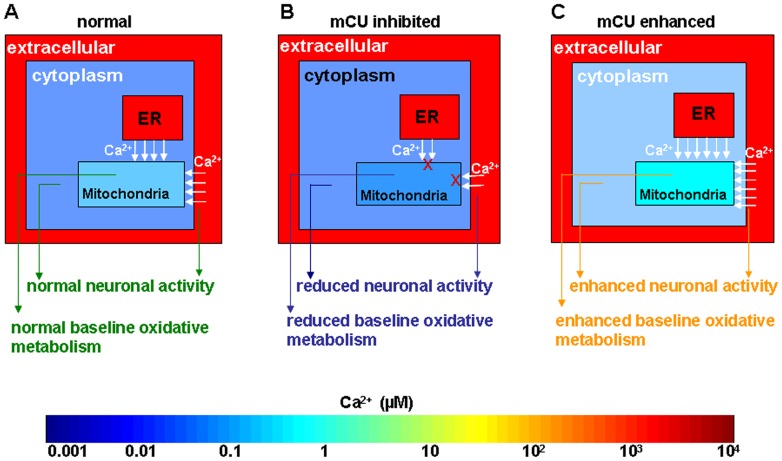
Multi-compartment evaluation of hypothetical Ca^2+^ ranges of in the brain tissue during spontaneous brain activity in A.normal, B. mCU inhibited and C. mCU enhanced conditions. Normal neuronal activity and baseline metabolism is maintained through the integrative role of mitochondrial Ca^2+^. During mCU inhibited conditions, diminished mitochondrial Ca^2+^ uptake reduces cytoplasmic Ca^2+^ influx with no overall change in the cytoplasmic Ca^2+^ levels leading to lesser neuronal activity and baseline metabolism. During mCU enhanced conditions, larger mitochondrial Ca^2+^ uptake enhances neuronal activity and baseline metabolism. We test these hypotheses through measurements of spontaneous neuronal activity, BOLD and CBF hemodynamic fluctuations. (ER-endoplasmic reticulum).

Normal and pathophysiological brain function is increasingly studied using Functional Magnetic Resonance Imaging (fMRI) which measure cerebrovascular correlates of neuronal function. But how the brain integrates neural activity (cell signaling events) and oxidative metabolic demand (an enzymatic activity) *in vivo* are not completely known. Considering the integrative role of mitochondrial Ca^2+^ buffering in “cell signaling” [4] and “metabolism” [5] and the primacy of the two processes in determining blood oxygen level dependent (BOLD) fMRI dynamics, mitochondrial Ca^2+^ homeostatic functions may have translational relevance through their impact on fMRI signals. This premise is supported by studies of spontaneous fluctuations of the mitochondrial enzyme cytochrome c oxidase activity (involved in oxidative metabolism) indicating that mitochondrial redox state fluctuates in a Ca^2+^ related manner while temporally preceding cerebral blood volume (CBV) fluctuations [6], [7]. Furthermore, Ca^2+^ also regulates mitochondrial dehydrogenases [5], [8] and co-varies with stimulation-induced BOLD signals [9]. Given the similar physiological origins of resting state and activity-evoked BOLD hemodynamic responses [1], and the dependence of activity-evoked hemodynamic responses on mitochondrial Ca^2+^ influx [2], we hypothesized that spontaneous neuronal activity and its cerebrovascular correlates will be altered in a mitochondrial Ca^2+^ uptake dependent manner. From *in vitro* studies of Ca^2+^ measurements in neuronal and glial cells [10], [11], [12], [13], [14], [15], [16], [17], [18], [19], [20], [21], [22], [23], we determined probable baseline Ca^2+^ ranges in multiple sub-cellular compartments within the brain tissue (Figure 1A). We hypothesized that reducing or enhancing mitochondrial Ca^2+^ uptake capacity would decrease or increase oxidative metabolism and spontaneous neuronal activities respectively in a cytoplasmic and mitochondrial-Ca^2+^ dependent manner (Figures 1B–C).

We tested the hypothesis using intact anesthetized rats where resting state spontaneous neural activities and CBF fluctuations were measured in conjunction with resting state fMRI-BOLD to infer intrinsic brain activities in normal and altered mitochondrial functional states. Mitochondrial Ca^2+^ uptake capacity was reduced by pharmacologically inhibiting the mitochondrial Ca^2+^ uniporter (mCU), (a primary mitochondrial inner membrane channel that transports Ca^2+^ ions from the cytoplasm into the mitochondria) with Ru360 treatment [24]. On the other hand, mitochondrial Ca^2+^ uptake capacity was increased by pharmacologically enhancing mCU activity with Kaempferol treatment [25]. Spontaneous neuronal activity diminished during mCU inhibition and increased during mCU enhancement. Despite opposite effects on the spontaneous neuronal activities, RSFC was diminished during both conditions. The results collectively establish that mitochondrial Ca^2+^ uptake capacities influence spontaneous neuronal activity and their hemodynamic fluctuations. While diminished mitochondrial Ca^2+^ uptake decreased neuronal activity and oxidative metabolism to reduce RSFC, enhanced mitochondrial Ca^2+^ uptake increased neuronal activity, oxidative metabolism and additionally altered output times of cortical neuronal populations to reduce RSFC. Hence perturbed mitochondrial Ca^2+^ uptake conditions (diminished or enhanced) may always lead to reduced intrinsic modes of brain functional connectivity determined by resting state fMRI.

## Materials and Methods

### Ethics Statement

All procedures were performed in accordance with protocols approved by the Yale University (no. 11194) and UMDNJ-New Jersey Medical School (no. 09009) Institutional Animal Care and Use Committees, in agreement with the National Institutes of Health Guide for the Care and Use of Laboratory Animals. All efforts were made to minimize suffering during initial surgery and throughout the experiments.

Experiments were conducted on urethane or α-chloralose anesthetized male rats (Sprague-Dawley; 250–300 g; n = 26; Charles River, Wilmington, MA) artificially ventilated with (70% N_2_O/30% O_2_). A femoral artery was cannulated (PE-50) for monitoring physiological parameters (pCO_2_, pO_2_, blood pressure) and a femoral vein was cannulated (PE-10) to administer D-tubocurarine chloride (initial 0.5 mg/kg; supplemental 0.25 mg/kg/h) and other substances including Ru360 and Kaempferol. Urethane anesthesia (1.3 g/kg, i.p) was used for the fMRI experiments and α-chloralose (40 mg/kg/h) for the electrophysiology experiments. Animals were surgically prepared under 2% isoflurane and switched to urethane or α-chloralose during the recording phase. Additional doses of urethane (0.13 g/kg, i.v.) were administered if necessary depending on the duration of the experiment. Adequate anesthesia was ensured by monitoring the pain response in blood pressure to an automated electrical (5 mA, 0.3 msec, 10 Hz, 1 sec) tail-pinch every ¼ hour in general but not during the resting scan measurements. pETCO_2_ (end tidal partial pressure of CO_2_) was maintained between 32–36 mmHg by appropriate ventilatory adjustments. The animal's core temperature was monitored and maintained at 37±0.5°C with a rectal probe and homeothermic blankets.

α-chloralose is the most commonly used anesthetic in rodent neuroimaging and physiology experiments from early studies showing that this agent preserved the robust hemodynamic and metabolic coupling to sensory stimulation [26]. Urethane, another widely used anesthetic minimally affected cardiovascular, respiratory and spinal reflexes, providing long-term stability and balanced actions on multiple neurotransmitter receptors [27]. Hence α-chloralose and urethane were suitably used depending upon the total experimental time with urethane preferred for the relatively longer functional Magnetic Resonance Imaging (fMRI) experiments. α-chloralose and urethane have similar effects on evoked potentials and hemodynamic response characteristics with subtle variations in evoked activity-induced responses to different stimulation frequencies [28]. Hence, during the resting state, spontaneous neuronal activities and their neurovascular coupling are expected to be linear and may not significantly differ between the two anesthetics. Though no major anesthetic confounds exist, differences in spontaneous high frequency bursts cannot be ruled out between the two anesthetics.

### Drug treatments

Ru360 was from EMD Biosciences San Diego CA and Kaempferol from Sigma Chemical Company, St Louis, MO. The first group of rats (n = 14; 7 for fMRI, 7 for electrical recording/LDF) were treated with Ru360 and the second group (n = 12; 7 for fMRI and 5 for electrical recording/LDF) were treated with Kaempferol. Control measurements were made temporally preceding the Ru360 or Kaempferol treatments. Physiological saline was used for the preparation of Ru360 working solution and 20% Dimethyl Sulfoxide (DMSO) in saline was used to prepare Kaempferol working solution. DMSO at the concentrations used has been shown to have no significant neuroactive effects [29]. In vitro studies suggest effective drug action on mitochondrial targets to take several minutes. Hence measurements within our *in vivo* experimental design with the mCU modulating drugs were generally performed 30 minutes after administration.

### Neuronal activity measurements

Rats were mounted on a stereotaxic frame (David Kopf Instruments, Tujunga, CA) placed on a vibration-free table inside a Faraday cage. The scalp and the *galea aponeurotica* were removed and small burr holes were drilled for insertion of high impedance tungsten microelectrodes (2–4 MΩ; FHC, Bowdoinham, ME) that measured neural electrical signals. Neural recordings were made from the forepaw region within the somatosensory cortex (S1_FL_). With reference to bregma and the sagittal midline plane the electrodes were placed in the coordinates 1 mm anterior, 4.4 mm lateral, ∼1 mm from dura within the S1_FL_ region. Somatosensory stimulation was delivered through electrical stimulation of the forelimb. Stimulus parameters consisted of 2 mA amplitude pulses of 0.3 msec duration where multiple pulses were separated by 333 ms. Electrical stimulation was delivered in an ON-OFF pattern of 30 sec pre-stimulus baseline measurement followed by stimulation for 30 sec followed by 30 sec post-stimulus baseline.

### Functional Magnetic Resonance Imaging (fMRI)

fMRI data were obtained on a modified 11.7T system with Varian (Varian Inc, Palo Alto, CA currently Agilent Technologies, Santa Clara, CA) spectrometer and custom built ^1^H surface coil (diameter 1.4 cm). Details of fMRI measurements are detailed elsewhere [30]. Resting state BOLD contrast images were acquired using echo-planar imaging (EPI) with sequential sampling and the following parameters: repetition time (TR)  = 1000 ms; echo time (TE)  = 13 ms; field of view (FOV)  = 2.56×2.56 cm^2^; image matrix  = 64×64, NR = 300. Five contiguous coronal slices with 2 mm slice thickness were selected over the region +2 mm anterior to and −6 mm posterior to the Bregma point. Anatomical images were obtained using gradient echo multi slice (GEMS) or fast spin echo multi slice (FSEMS) contrast sequences in 128×128 matrix and FOV  = 2.56 cm. In the anesthetized rat model, different gradient amplitudes and pulsing rates do not alter blood pressure and electrical recordings outside the magnet (on the bench) and inside the magnet (during an fMRI scan).

### Laser Doppler flowmetry (LDF)

In all rats that underwent electrical neuronal activity measurements, baseline CBF fluctuations were simultaneously measured in a high temporal resolution (200 Hz) using a laser Doppler probe (Oxford-Optronix, Oxford, UK). The CBF probe was inserted into the cortex adjacent to the neuronal recording electrode within the somatosensory region.

### Data analysis

Neuronal activity measurements in the form of multi unit activity (MUA) and local field potentials (LFP) were measured both during the resting state and somatosensory stimulation states. Electrophysiological signals obtained were digitized at 20 kHz and actively filtered to LFP and MUA signals (Krohn-Hite Corp., Brockton, MA) by splitting the electrical signals into low (<150 Hz) and high frequency (0.4–10 kHz) frequency bands using a Butterworth filter (24dB/oct attenuation). Electrophysiological data were simultaneously recorded using the Spike2 software (Cambridge Electronic Design, Cambridge, UK).

**Figure 2:**
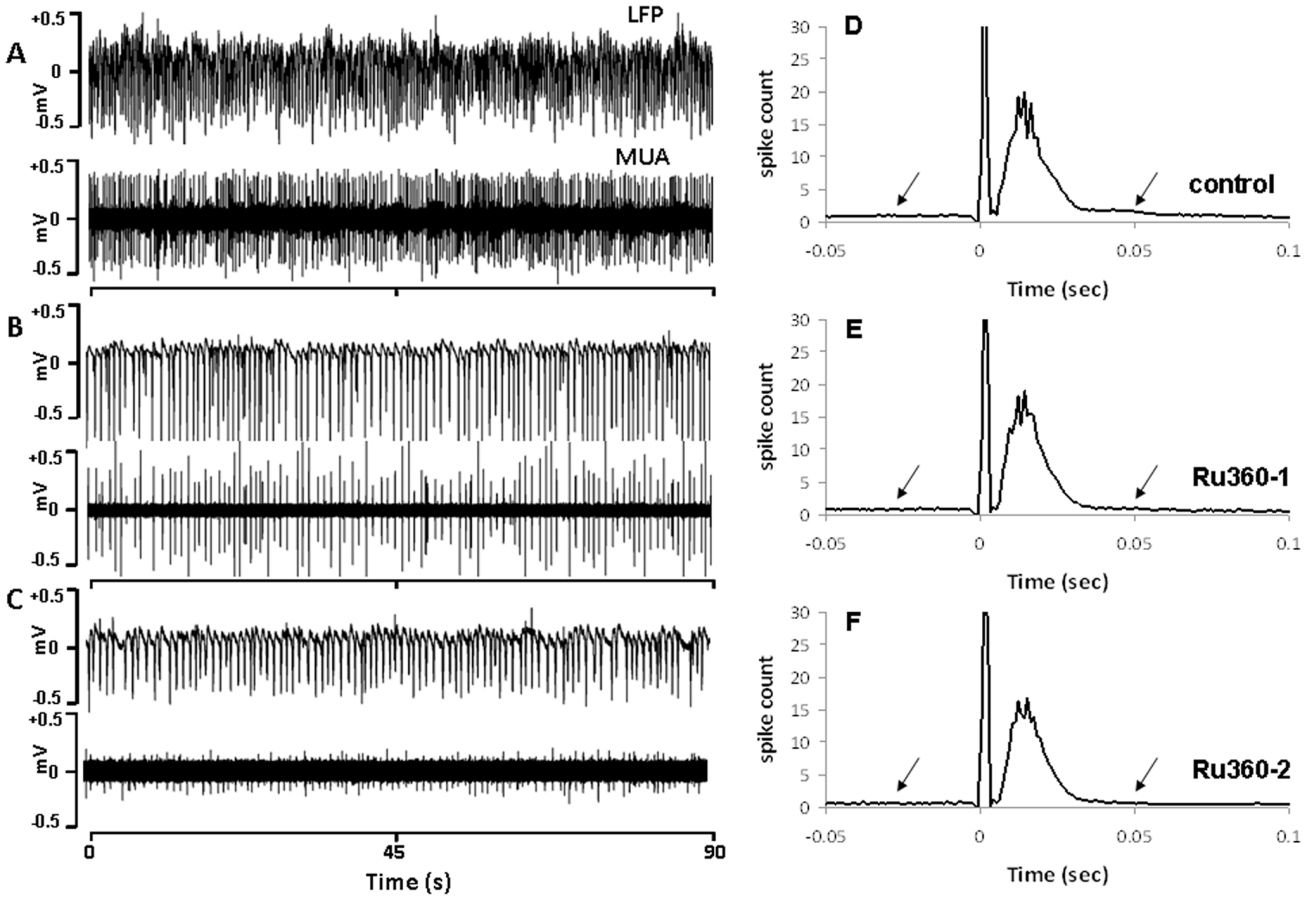
Ru360 in a dose-dependent manner (Ru360 dose-1 = 120 µg/Kg; dose-2 = 240 µg/Kg), decreased the resting state spontaneous electrical activity in the somatosensory cortex. Typical traces of LFP and MUA in **A.** control, **B.** 120 µg/kg Ru360 and **C.** µg/kg Ru360 treated conditions. **D–F.** Peristimulus time histograms (PSTH) during evoked somatosensory stimulation estimated from 60–90 trials over 7 animals in the Ru360 treatment group indicate dose-dependent reduction in the resting-state pre-stimulus and post-stimulus baselines (indicated by the arrows). As indicated in panel **E.** reduced stimulus-evoked spiking of multiple units becomes apparent at higher doses of Ru360 treatment. Spike counts decayed exponentially from the respective peak times to 0.05 sec (R^2^>0.95; Table 1). Multiple units spiked significantly faster after arrival of the evoked stimulus both during Ru360 (Table 1). Spike counts at 0-sec are stimulus artifacts.

**Figure 3:**
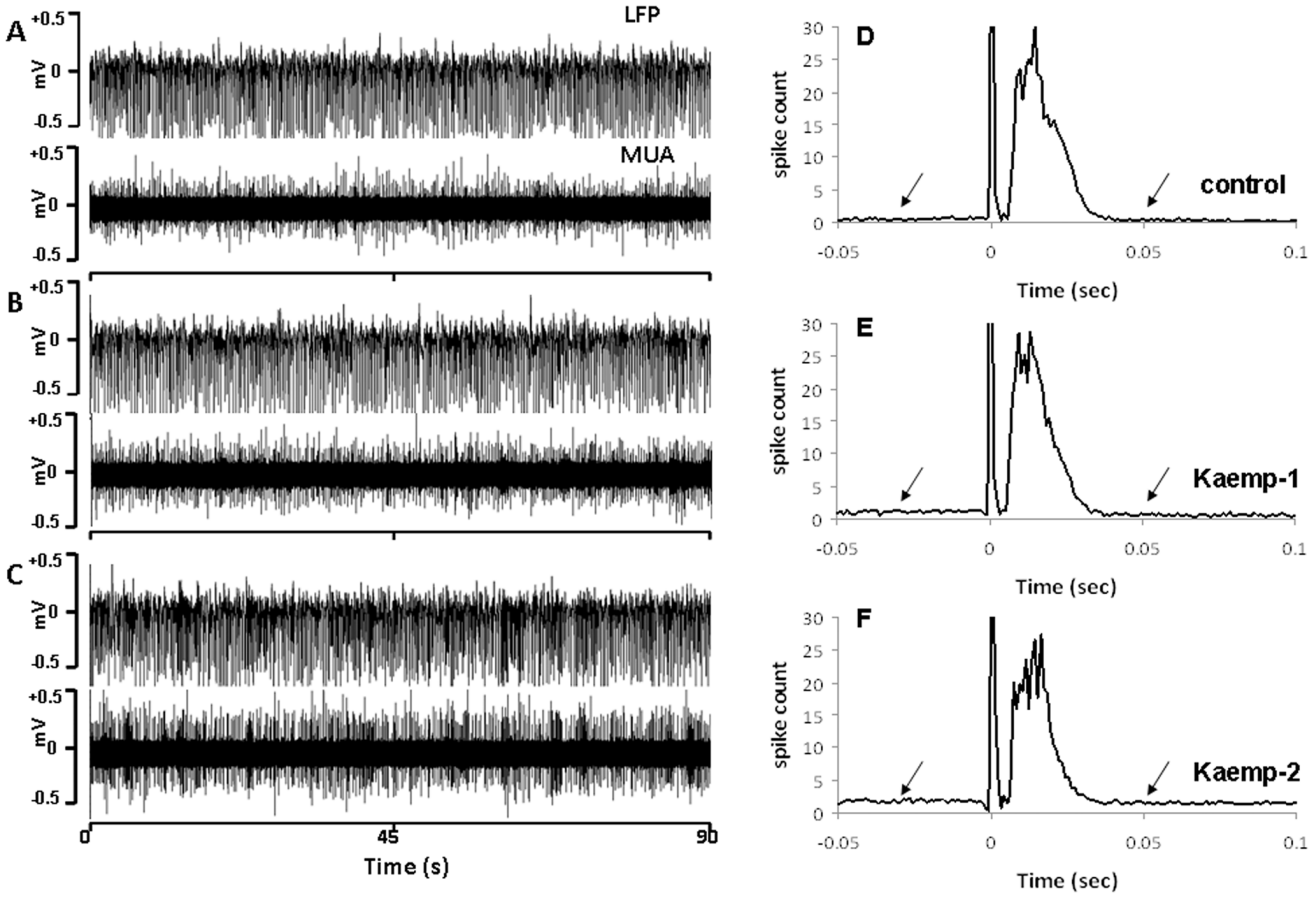
Kaempferol in a dose-dependent manner (Kaempferol dose-1 = 1 mg/kg; dose-2 = 2 mg/kg) increased the resting state spontaneous electrical activity in the somatosensory cortex. Typical traces of LFP and MUA in **A.** control, **B.** 1 mg/kg Kaempferol and **C.** 2 mg/kg Kaempferol. **D–F.** Peristimulus time histograms (PSTH) during evoked somatosensory stimulation estimated from 30–60 trials over 5 animals in the Kaempferol treatment group indicate dose-dependent enhancement in the resting-state pre-stimulus and post-stimulus baselines (indicated by the arrows). As indicated panels **E** and **F.** increased stimulus-evoked spiking of multiple units becomes apparent with Kaempferol treatment. Spike counts decayed exponentially from the respective peak times to 0.05 sec (R^2^>0.95; Table 1). Multiple units spiked significantly faster after arrival of the evoked stimulus both during Kaempferol treatment (Table 1). Spike counts at 0-sec are stimulus artifacts.

For the resting state neuronal activity measures, spike activity was extracted from MUA time series with wavelet spike sorting and super-paramagnetic clustering algorithms [31]. Data sections with 3 minute lengths were selected for analysis. Spiking frequency was calculated in 1 sec bins, and subsequently averaged in the selected time series. LFP activity was estimated using the root-mean-square (RMS) calculation. While there are many approaches to simplify LFP time courses (e.g. filtering, down-sampling), we selected the RMS approach to avoid loss of LFP signal power as the RMS approach rectifies the signal before binning. As originally introduced on MUA signals [32] containing negative and positive components (both of which are important parts of the signal) the RMS calculation was adequate for LFP signals too, because our LFP signals had both negative and positive components (Figures 2A–C and 3A–C). Briefly, the raw data was squared, averaged for 1 sec bins, after which the square root was calculated. The mean RMS was estimated for every data block.

Task-evoked neural activity was analyzed using the peristimulus time histogram (PSTH; Figures 2D–F and 3D–F). PSTH were created as the number of identified spikes from the MUA time series in 1 msec bins. Spikes within a 50 msec interval pre-stimulus and 100 msec after the stimulus were counted within each 1 msec bin to estimate the frequency histogram of 150 bins. While the total interval between stimuli were 333 ms, the number of spikes didn't change after 100 ms. Decay of spiking activity of neuronal populations from the PSTH was estimated from the peak time to 0.05 sec after least square fitting to an exponential function: Y = Y_0_ e^−t/τ^. Time constant ‘τ’ after treatment were compared to the respective control group and considered significantly different when there were no overlaps in the 95% confidence intervals of the fit between the two conditions.

R-fMRI data were analyzed using AFNI [33]. After co-registering with the respective anatomical images from each animal, functional EPI images were corrected for motion within-scan using a rigid-body volume registration in AFNI (3dvolreg) with the 10^th^ BRIK as the base. In all within-scan corrected datasets from each subject, motion between-scans were further corrected using the within-scan corrected 10^th^ BRIK from the first scan dataset as the base. Temporal standard deviations of the R-fMRI time series estimated before and after detrending showed differences <0.5%, indicating that scanner drifts were minimal. Hence only the data considered for temporal correlation analysis were detrended to remove quadratic trends, whereas no detrending was carried out during power spectral analyses in order to retain very low frequency physiological noise structures. As the animals were artificially ventilated at a rate of 1 cycle/sec approximating the sampling TR of 1 sec, inter-subject and temporal variations in respiratory aliasing were greatly minimized. Due to coupling of respiration and apparent motion at the ultra high field (11.7 T) used in this study, motion correction within-scan has been shown as an effective method in removing physiological noise arising from respiration [34] and was adopted here without any further signal regression of any kind. To complete the removal of physiologic noise a low pass filtering (0.1 Hz cutoff) of the R-fMRI time series was performed to eliminate remaining respiratory and cardiac contributions to the noise during estimation of RSFC.

**Figure 4:**
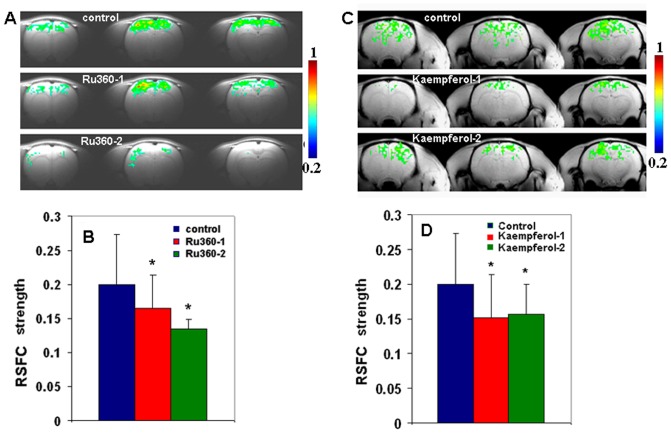
mCU modulation impacted the resting state functional connectivity (RSFC). Inhibition of mCU activity with Ru360 (dose-1 = 120 µg/kg; dose-2 = 240 µg/kg) reduced **A.** spatial extent of the mean resting state functional connectivity (RSFC) determined using cross correlation of seed voxels from the somatosensory cortex region of interest with all other voxels within the brain in typical animals and reduced **B.** mean RSFC strength in the cortex over 7 animals within the Ru360 treatment group. Enhancement of mCU activity with Kaempferol (dose-1 = 1 mg/kg; dose-2 = 2 mg/kg) reduced **C.** spatial extent of the mean resting state functional connectivity (RSFC) in typical animals and reduced **D.** mean RSFC strength in the cortex over 7 animals within the Kaempferol treatment group. Color scale represents the correlation coefficient (r) values above threshold ≥0.2; uncorrected P<0.0001. Voxels with r≥0.2 were deemed active and corrected with a cluster-size of 10 voxels to control for type-I error. Significantly different compared to control *P<0.01; one-way ANOVA with post-hoc Tukey's HSD test. Error bars represent the group standard deviation.

**Figure 5:**
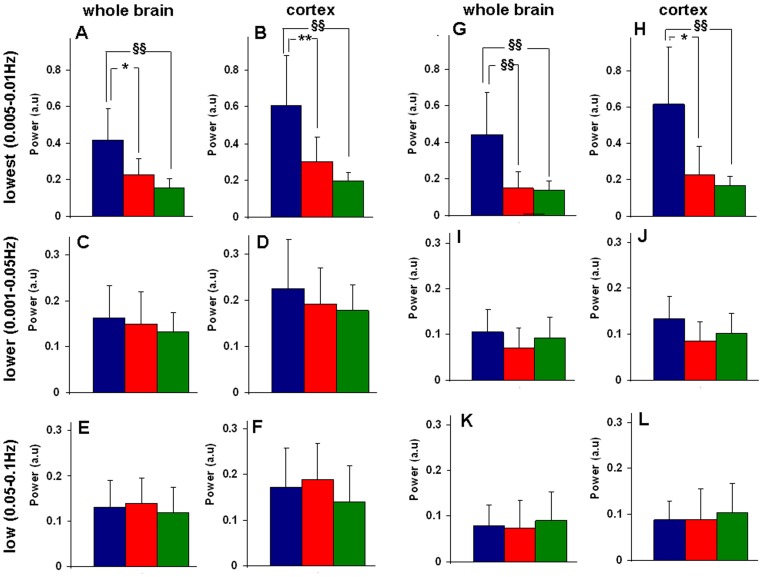
mCU modulation impacted spectral power of the low frequency (<0.1 Hz) R-fMRI BOLD fluctuations in the whole brain and cortex during A–F. Ru360 and G–L. Kaempferol treatment. R-fMRI spectral power were analyzed in three different frequency bands namely lowest (0.005–0.01 Hz), lower (0.01–0.05 Hz) and low (0.05–0.1 Hz) respectively. Inhibition of mCU activity with Ru360 (dose-1 = 120 µg/kg; dose-2 = 240 µg/kg) reduced the spectral power in the lowest band for **A.** the whole brain and **B.** cortex. No significant effect was observed in the **C, D.** lower band and **E, F.** low band over 7 animals from the Ru360 treated group. Enhancement of mCU activity with Kaempferol (dose-1 = 1 mg/kg; dose-2 = 2 mg/kg) reduced the spectral power in the **G, H.** lowest band. No significant effect was observed in the **I, J.** lower and **K, L.** low band over 7 animals from the Kaempferol group. Significantly different compared to control ^§§^P<0.001, *P<0.005, **P<0.02; Kolmogorov-Smirnov test. Blue:control; red:dose-1; green:dose-2. Error bars represent the group standard deviation.

As the objective of this study was to compare drug effects on the resting state functional connectivity strength in an apriori (somatosensory) region of interest (ROI) with maximum sensitivity, creation of average time series from a large seed ROI (as usually done in standard human brain analyses) was not followed. Mean time courses generated from finite seed ROIs would correlate at a high rate in regions that contribute to the mean seed time series, thus reducing sensitivity. With the apriori (somatosensory) ROI defined from the rat atlas in stereotaxic co-ordinates [35], [36], seed voxels were randomly selected for the determination of RSFC maps. RSFC maps were determined after cross correlating the filtered time series from a seed voxel with all other voxels within the brain [35]. 6 seed voxels were randomly selected within the somatosensory ROI (3 each from either hemisphere) to generate 6 correlation coefficient ‘r’ maps (Figure S1). This process was repeated across 3 trials of every experimental condition leading to 18 RSFC maps for each experimental condition per animal. All RSFC maps were subsequently z-transformed (z = 0.5*log[(1+r)/(1−r)]) by estimating the arctanh of ‘r’ on a voxel-wise basis and averaged [37], [38]. The averaged z-map was transformed by estimating the tanh of the z-values to obtain the averaged ‘r’ map after applying a threshold of r≥0.2 to determine the mean RSFC map for each treatment per animal (Figures 4A and 4C). RSFC strength per animal was determined by averaging the absolute value of the correlation coefficients across voxels within the somatosensory ROI from the averaged ‘r’ map without any threshold. The estimated RSFC strength per animal was averaged across all animals within the group to determine the group-RSFC strength for each treatment (Figures 4B and 4D). The ‘r’values within subjects were converted to a z-score to avoid bias in the voxel statistic after averaging to obtain an accurate mean RSFC map in every subject. On the other hand, no voxel-wise statistic was estimated across animals since this study did not cover the whole brain but the cortical and neighboring areas. As registration to a standard atlas was not performed across animal subjects, a subject-wise average of the correlation coefficient value obtained from the cortical ROI of each animal was considered for RSFC strength estimation at the group level. Power spectra were estimated by Fast Fourier Transforming temporal signals in a voxel-wise manner. Spectral power within three frequency bands in the low frequency region (<0.1 Hz) were determined consisting of the lowest (0.005–0.01 Hz), lower (0.01–0.05 Hz) and low bands (0.05–0.1 Hz) respectively (Figure 5). Average spectral power at selected frequency bands were color-encoded on a voxel-wise basis within the corresponding EPI images to visualize brain areas that exhibited fluctuations at the respective frequency bands (Figure S2 and S3).

**Figure 6:**
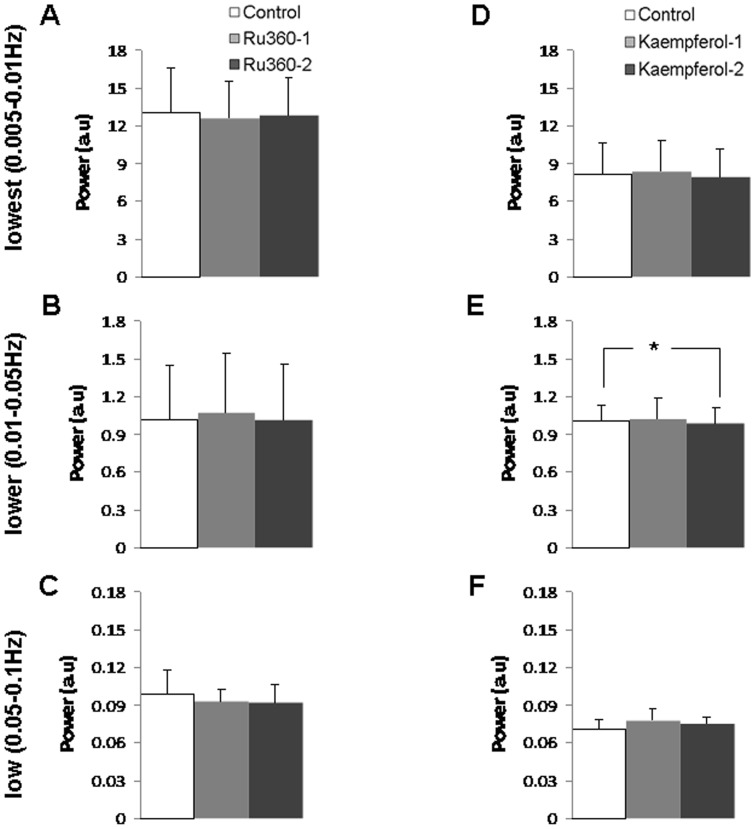
mCU modulation did not have a significant impact on the spectral power of the low frequency (<0.1 Hz) cortical CBF fluctuations. Inhibition of mCU activity with Ru360 (dose-1 = 120 µg/kg; dose-2 = 240 µg/kg) did not affect the CBF spectral power in three different low frequency bands **A.** lowest (0.005–0.01 Hz) **B.** lower (0.01–0.05 Hz) and **C.** low (0.05–0.1 Hz). Enhancement of mCU activity with Kaempferol (dose-1 = 1 mg/kg; dose-2 = 2 mg/kg) reduced the CBF spectral power only in the **E.** lower (0.01–0.05 Hz) frequency band with no significant effect on the **D.** lowest (0.005–0.01 Hz) and **F.** low (0.05–0.1 Hz) frequency bands. Significantly different *P<0.05; Kolmogorov-Smirnov test. Error bars represent the group standard deviation.

Laser Doppler time series obtained from the flow probe at high temporal resolution were analyzed in the frequency domain using MATLAB. Spectral power of the low frequency (<0.1Hz) CBF fluctuations were estimated in three frequency bands during the normal and mCU modulated conditions (Figure 6).

Post-hoc Tukey's HSD test was used to further explore any significant effects revealed by ANOVA. For the power spectral data which were not normal, the non-parametric Kolmogorov-Smirnov test was used. P<0.05 was considered significant.

## Results

Spontaneous neuronal activity measurements were made during the resting state in the absence of any evoked stimulus. mCU inhibition with Ru360 significantly diminished the spontaneous neuronal activity across typical animals (Figure 2A–C). After the resting state measurements, animals underwent forepaw stimulation during which the electrical measurements were continued. Peristimulus time histogram (PSTH) of evoked stimulus-induced MUA activity changes was estimated from multiple measurements over all animals within the Ru360 treatment group (Figures 2C–E). As indicated by the arrows, resting-state (pre- and post-stimulus baseline) spiking of multiple units reduced after the higher dose of Ru360 treatment (Figure 2E). From the pre-stimulus data sections of 3 minute lengths (described in the methods), baseline spiking frequencies were estimated to be 10.61±4.5 Hz in the control (prior to treatment), which decreased to 10.14±5.25 Hz and 8.60±5.34 Hz respectively after treatment with first and second doses of Ru360. Spiking frequency decrease during the second dose of Ru360 was significantly different compared to controls (P<0.05; ANOVA, post-hoc Tukey's HSD test). Timing of neural responses after evoked stimulus was assessed by estimating the time of peak neural activity (Table 1). Decay of neural responses from the respective time of peak activity to 0.05 sec was estimated after fitting a model exponential function (goodness of fit, R^2^>0.95; Table 1). Multiple units spiked significantly faster after arrival of the evoked stimulus during Ru360 treatment (Table 1).

**Table 1:** Exponential fit parameters of the spike count decay from the peak time to 0.05 sec of the peristimulus time histogram (PSTH) estimate in the Ru360 and Kaempferol treated rats.

	Ru360			Kaempferol		
	control	dose-1	dose-2	control	dose-1	dose2
**no.of measures**	178	107	88	29	21	29
**peak time (msec)**	14.3	14.3	15.3	14.3	13.3	16.3
**peak spike count**	20.02	19.01	16.86	29.97	28.81	27.48
**amplitude (Y_0_)**	75.42 (57; 94)	114 (94; 134)	133.2 (111; 155)	136.1 (100; 172)	171.9 (144; 200)	441.2 (280; 602)
**τ (msec)**	9.8 (8.7; 11.2)	8.0 (7.5;8.7)	7.3* (6.9; 7.9)	8.9 (7.9; 10.0)	7.7* (7.2; 8.3)	5.7* (5.1; 6.3)
**goodness of fit (R^2^)**	0.94	0.98	0.99	0.95	0.98	0.96

Values in parenthesis are the lower and higher confidence values for a 95% confidence interval; significantly different compared to the respective control group * P<0.05.

mCU enhancement with Kaempferol significantly enhanced spontaneous neuronal activity across typical animals (Figure 3A–C). After the resting state measurements, animals underwent forepaw stimulation during which the electrical measurements were continued. Peristimulus time histogram (PSTH) of evoked stimulus-induced MUA activity changes was estimated from multiple measurements over all animals within the Kaemferol treated group (Figures 3C–E). As indicated by the arrows, resting-state (pre- and post-stimulus baseline) spiking of multiple units reduced with Kaempferol in a dose-dependent manner (Figure 3D–E). From the pre-stimulus data sections of 3 minute lengths (described in the methods), baseline spiking frequencies were estimated to be 10.67±1.06 Hz in the control, which significantly increased to 13.41±1.69 Hz (P<0.01; ANOVA, post-hoc Tukey's HSD test) and 15.51±3.14 Hz (P<0.05; ANOVA, post-hoc Tukey's HSD test) respectively after treatment with first and second doses of Kaempferol. Timing of neuronal responses after the evoked stimulus was assessed by estimating the time of peak neural activity (Table 1). Decay of neuronal responses from the respective time of peak activity to 0.05 sec was estimated after fitting a model exponential function (goodness of fit, R^2^>0.95; Table 1). Multiple units spiked significantly faster after arrival of the evoked stimulus with Kaempferol treatment (Table 1). These results collectively indicate that inhibition of the mitochondrial Ca^2+^ influx process decreased whereas enhancement of the same increased neuronal activity.

RSFC was assessed using the temporal characteristics of the R-fMRI BOLD signal from seed voxels within the somatosensory cortical region. Correlation coefficients were determined throughout the brain after cross correlation of a seed voxel time series with voxels across the entire brain. Six RSFC maps were generated from six randomly selected seed voxels from either hemisphere for every experimental condition in each animal (Figure S1). Typical RSFC maps were bilateral and reproducible for every random seed selection (from both hemispheres) extending towards homologous regions on the opposite hemisphere (Figure S1). Figure 4A shows the mean RSFC maps from a typical animal before and after treatment with Ru360. Typical RSFC (Figure S1) and mean RSFC maps covered most somatosensory regions on either hemisphere [39], [40], [41] and decreased in spatial extent after treatment with Ru360. RSFC strength was determined by spatially averaging the absolute values of correlation coefficients across voxels within the cortical ROI in the average RSFC map from each animal. Figure 4B shows the RSFC strength from all animals within the group before and after treatment with Ru360. RSFC strength in the cortical ROI did not change significantly with 120 µg/kg Ru360 but reduced by 32% with 240 µg/kg of Ru360 (Figure 4B). Kaempferol-treated rats were analyzed in a similar manner. RSFC extended towards homologous regions on the opposite hemisphere for each random seed voxel selection (Figure S1). Figure 4C shows the mean RSFC map from a typical animal indicating a decrease in the spatial extent after treatment with Kaempferol. The correlation threshold of r≥0.2 represented signal with high probability, however average RSFC strength varied below the threshold of 0.2 (Figure 4). This was due to the consideration of all correlation coefficients within the cortical ROI for averaging which, depending upon the subject and drug treatment included a varying number of voxels below 0.2 contributing to the RSFC strength. RSFC strength was determined by spatially averaging the absolute values of correlation coefficients across voxels within the cortical ROI in the mean RSFC map from each animal. RSFC strength in the cortical ROI significantly decreased by 25% after treatment with 1 mg/kg Kaempferol with no further decrease with 2 mg/Kg Kaempferol (Figure 4D).

In a frequency-domain analysis, power spectra of the R-fMRI BOLD signals were determined on a voxel-wise basis and the low frequency spectral power was estimated in three different bands during control and mCU modulated conditions. Whole brain averaged-low frequency spectral power was the largest in the cortex and thalamus (Figures S2B–D). Spectral power in the lowest band (0.005–0.01 Hz) decreased considerably over all brain regions including the cortex with Ru360 treatment in typical (Figures S2) and over all animals (Figure 5A and 5B). There was no significant change in spectral power in the lower (0.01–0.05 Hz) and low bands (0.05–0.1 Hz) (Figures 5C–D and Figure 5E–F). However, the lowest band spectral power decreased in the whole brain by 48% during treatment with 120 µg/kg of Ru360 and by 56% with 240 µg/kg of Ru360 (Figure 5A). A dose dependent monotonous decrease in the cortex with a 50% and 68% decrease was also observed (Figure 5B). During Kaempferol treatment, spectral power in the lowest band (0.005–0.01 Hz) decreased considerably over the whole brain and cortex in typical (Figure S3) and over all animals (Figure 5G and 5H). There was no significant decrease in spectral power in the lower (Figure 5I–J) and lowest bands (Figure 5G–H). However, the lowest band spectral power decreased in the whole brain by 58% with 1 mg/Kg and by 60% with 2 mg/Kg of Kaempferol (Figure 5G). In the cortical ROI, the lowest band spectral power decrease was 60% and 66% respectively after treatment with the two doses of Kaempferol (Figure 5J).

In rats that underwent electrophysiological measurements to estimate neuronal activity, simultaneous baseline CBF fluctuations were measured with a high temporal resolution using a laser Doppler probe inserted into the cortex adjacent to the electrode within the somatosensory region. Laser Doppler time series were analyzed in the frequency domain similar to R-fMRI BOLD signals. Spectral power of the low frequency (<0.1 Hz) CBF fluctuations were estimated during normal and mCU modulated conditions. Ru360 treatment had no significant effect on the spectral power from all frequency bands (Figure 6A–C) whereas Kaempferol significantly reduced the lower band (0.01–0.05Hz) spectral power (Figure 6E) with no significant effect on the lowest and low band spectral power (Figure 6D and 6F).

## Discussion

During normal conditions spontaneous neural activity is enabled by the function of receptor operated and voltage-gated Ca^2+^ channels leading to increased cytoplasmic and mitochondrial Ca^2+^. When mCU is inhibited, cells lack the mitochondrial Ca^2+^ sink, leading to reduced glutamate receptor-mediated intracellular Ca^2+^ influx [13], [42]. Reduced glutamate receptor activity during inhibition of mitochondrial Ca^2+^ influx can occur through accumulating Ca^2+^ within cytoplasmic microdomains leading to N-methyl D-aspartate (NMDA) channel desensitization [13], [15], [16], which may limit further cytoplasmic Ca^2+^ influx. Hence an unchanged overall cytoplasmic and decreased mitochondrial Ca^2+^ levels may constitute the new baseline equilibrium of decreased spontaneous activity of neuronal populations which returned back to the same new equilibrium after evoked neuronal activation during mCU inhibition (Figure 2). As spontaneous neuronal activity influences resting state BOLD fluctuations [43], [44], [45], the observed decrease in BOLD fluctuations (Figures 5A and 5B) and RSFC (Figures 4A and 4B) raises the possibility that diminished mitochondrial Ca^2+^ uptake capacity may lead to decreases in spontaneous neuronal activity and diminish BOLD fluctuations and RSFC. Additionally a relatively lesser mitochondrial matrix Ca^2+^ (Figure 1B) and its fluctuations in the resting state will occur during mCU inhibition. Lacking sufficient Ca^2+^ dependent acceleration of the matrix dehydrogenases [5], mitochondria may not sustain normal levels of oxidative metabolism and its fluctuations. Thus the extent of mitochondrial Ca^2+^ influx may modulate spontaneous neuronal activity through both cytoplasmic and mitochondrial Ca^2+^ levels to impact the hemodynamic BOLD fluctuations. However, altered mitochondrial Ca^2+^ homeostasis during mCU inhibition that decreased spontaneous neuronal activity in a subtle manner was not robust enough to drive measurable changes in CBF fluctuations. These results indicate that CBF and BOLD fluctuations may possibly decouple during diminished mitochondrial Ca^2+^ uptake capacities.

Enhanced mCU activity can lead to larger cytoplasmic and mitochondrial Ca^2+^ loads (Figure 1C). The excess matrix Ca^2+^ will be actively cycled out into the cytoplasm via the mitochondrial Na^+^/Ca^2+^ and H^+^/Ca^2+^ exchangers leading to a relatively larger cytoplasmic Ca^2+^ levels than normal conditions. During this state, spontaneous synaptic depolarization translate into higher probabilities of neuronal action potentials, augmenting subsequent synaptic depolarization and increasing the collective cortical excitability. The results during Kaempferol treatment increased spontaneous neuronal activity (Figure 3) supporting our hypothesis. Baseline neuronal activity states during mCU enhancement reached a new equilibrium with raised levels of spontaneous neuronal activities pre- and post-stimulus (Figures 3D–F). With substantially shortened evoked output timing of the cortical neuronal populations (Table 1), enhanced mCU activity may also significantly alter cortical synchrony and information transfer. Considering the dose-dependent increase in spiking frequency (Figure 3C–E) and speed (Table 1) accompanied by a saturated decrease in RSFC (Figure 4C–D) and low frequency BOLD spectral power (Figure 5G–L), the impact of neuronal signaling alone is insufficient to explain the saturated RSFC decrease. Possibilities exist where enhanced mitochondrial matrix Ca^2+^ may metabolically influence BOLD fluctuations. During enhanced mCU activity, spontaneous baseline neuronal activity would increase mitochondrial matrix Ca^2+^ over and above the levels expected normally (Figure 1C). With abundant matrix Ca^2+^, mitochondria would be in a position to accelerate oxidative metabolism in a Ca^2+^ dependent manner to match the new equilibrium of enhanced baseline oxidative energy consumption rate [5]. The resulting larger mitochondrial Ca^2+^ load will need to be actively cycled out into the cytoplasm by the mitochondrial Na^+^/Ca^2+^ and H^+^/Ca^2+^ exchangers using energy from the mitochondrial membrane potential (ΔΨ_m_). Global cytoplasmic Ca^2+^ homeostasis would be maintained by the plasma membrane Na^+^/Ca^2+^ exchanger and plasma membrane Na^+^/K^+^-ATPase activities, but working at a higher level than normal and consuming relatively larger adenosine triphosphate (ATP) dependent energy. It is very likely that the increased baseline metabolic demand triggered by larger spontaneous neuronal activity, synergized by higher mitochondrial matrix Ca^2+^ during the first dose of Kaempferol itself was sufficient to maximally inhibit resting state BOLD fluctuations so that further neuronal signaling modulations at higher Kaempferol doses (Figure 2C, 2D and Table 1) had no further impact on BOLD fluctuations. While increased Ca^2+^ loads in the cytoplasm and mitochondrial matrix may combine to increase spontaneous neuronal activity and reduce BOLD fluctuations, mitochondrial matrix Ca^2+^ and its cycling maybe more potent in impacting BOLD fluctuations.

Altered mitochondrial Ca^2+^ homeostasis during mCU enhancement that monotonously increased spontaneous neuronal activity may have been sufficiently robust at the higher Kaempferol dose where CBF fluctuations start to diminish (Figures 6D–F). These results indicate that large spontaneous neuronal activity changes are required to alter baseline CBF fluctuations compared to BOLD. Distinct neuronal-CBF and neuronal-BOLD relationships have been characterized by earlier studies and attributed to a non-linear relationship between neuronal activity and the relevant hemodynamic measure [46]. BOLD activity has been observed to change more rapidly than neuronal responses for small neuronal activity changes [47], but very different for CBF (measured by laser Doppler). CBF does not increase till a threshold of neuronal activity is reached thereafter which CBF increases in proportion to neuronal activity [48]. As observed by our results during mCU inhibition and enhancement, a relatively larger neuronal activity change threshold seem to be needed for significant changes in CBF fluctuations compared to BOLD. As glial Ca^2+^ changes can additionally affect vasomotion [49], [50] and prolong BOLD responses [9], there is a possibility that glial systemic effects maybe minimal at lower neuronal activity and metabolic thresholds to influence BOLD and CBF fluctuations differently, which may disappear at higher neuronal activity and metabolic levels.

Spontaneous cortical neuronal oscillations are phylogenetically preserved indicating that they are functionally relevant to brain function [51]. R-fMRI BOLD fluctuations correlate with underlying spontaneous cortical neuronal activity [43], [44], [45], [52] and may represent fluctuations in neuronal oxidative metabolism since they are similar to Ca^2+^ related mitochondrial redox state fluctuations [5]. Though mitochondrial functions in integrating Ca^2+^ signals seems to be universal, observed in several cell types of the brain (including neurons and glia) and other organ systems including the heart and liver, properties of specific functional networks within the brain can be heterogeneous. This heterogeneity may depend upon mitochondrial structural or functional diversity, participating cellular types and timing of spontaneous calcium changes and concentrations. As Ca^2+^ changes in glial networks during neuronal activity can modulate BOLD signal dynamics [9], the degree of the observed effects of mitochondrial functional state on RSFC within the cortical network may vary from other networks of the brain. Though varying in degree, the universal mechanism of mitochondrial Ca^2+^ integration can be expected to be conserved within various functional networks.

Contrasting bioenergetic states induced by deficient or enhanced mitochondrial Ca^2+^ influx resulted in contrasting neuronal activity levels with consequent loss of coherent activity within the somatosensory functional network. The perturbed mitochondrial states in the present study have translational relevance to human neuropathologies. Neurobiological evidence indicate decreased mitochondrial Ca^2+^ uptake in chronic neuropathological conditions such as aging and age-related diseases [53], [54], [55], [56], [57], [58]. These chronic neuropathologies are mostly accompanied by decreased RSFC determined from fMRI studies of aged humans and age-related neuropathological patients [59], [60], [61], [62]. On the other hand, neurobiological evidence indicate increased mitochondrial Ca^2+^ uptake with hyperexcited neuronal pathways [63], [64], [65] and increased baseline metabolism [8], [66], [67], [68], [69] in acute states after brain trauma and epilepsy. These neuropathological states that contrast bioenergetically with chronic neuropathological states such as aging, still show decreased RSFC as observed from fMRI studies of acute brain trauma or pediatric epileptic patients [70], [71]. Our results lead to the translational hypothesis that impaired mitochondrial Ca^2+^ uptake capacity reduces RSFC in aging humans and larger mitochondrial Ca^2+^ uptake reduces RSFC in brain trauma and pediatric epileptic patients. As mitochondrial dysfunction alters baseline neuronal activity and RSFC in unique ways, specific neurological disease amenable to mitochondrial therapy can be monitored through clinically relevant resting state fMRI biomarkers integrated with high resolution neural recordings (e.g., EEG in humans) [72].

## Supporting Information

Figure S1 **Typical resting state functional connectivity (RSFC) maps during mCU modulation.** Seed voxels within the somatosensory cortex region of interest on either hemisphere were chosen at random and cross correlated with all voxels within the brain to generate six RSFC maps for each experimental condition. **A–C.** show the representative maps (correlation coefficient threshold of 0.2 corresponding to a P<0.0001; corrected for multiple comparisons) for control and Ru360 treated conditions (Ru360 dose-1 = 120 µg/kg; dose-2 = 240 µg/kg), **E–F.** show the same for control and Kaempferol treated (Kaempferol dose-1 = 1 mg/kg; dose-2 = 2 mg/kg). The seed voxel shows the highest correlation coefficient value in the maps (indicated by arrows). Six such correlation coefficient maps were averaged to determine the mean RSFC map for each experimental condition in an animal. A similar procedure was repeated for all animals.
(TIF)

Figure S2 **mCU modulation reduced the spectral power of the low frequency (<0.1 Hz) R-fMRI BOLD fluctuations.** Inhibition of mCU activity with Ru360 (dose-1 = 120 µg/kg; dose-2 = 240 µg/kg) reduced the **A.** average power spectra (mean across voxels from the whole brain) in typical animals. R-fMRI spectral power were analyzed in three low frequency bands namely, lowest (0.005–0.01 Hz), lower (0.01–0.05 Hz) and low (0.05–0.1 Hz) indicated by different shades of blue color in panel **A. B.** the lowest band spectral power decreased during control and treatment with 120 µg/Kg and 240 µg/Kg Ru360 respectively. No significant change in the spectral power was observed between control and Ru360 treated states in the **C.** lower and **D.** low bands.
(TIF)

Figure S3 **mCU modulation reduced the spectral power of the low frequency (<0.1 Hz) R-fMRI BOLD fluctuations.** Enhancement of mCU activity with Kaempferol 1 mg/kg and 240 mg/kg Kaempferol reduced the **A.** average power spectra (mean across voxels from the whole brain) in typical animals. R-fMRI spectral power were analyzed in three low frequency bands namely, lowest (0.005–0.01 Hz), lower (0.01–0.05 Hz) and low (0.05–0.1 Hz) indicated by different shades of blue color in panel **A. B.** the lowest band and **C.** lower band spectral power decreased during treatment with 1 mg/kg and 2 mg/kg Kaempferol respectively with no effect on the **D.** low band spectral power.
(TIF)
