# Supplementary figures and images for: Mitochondrial Functional State Impacts Spontaneous Neocortical Activity and Resting State fMRI
Source: PLoS One. 2013 May 1;8(5):e63317. doi: 10.1371/journal.pone.0063317 (PMC3641133; doi:10.1371/journal.pone.0063317)

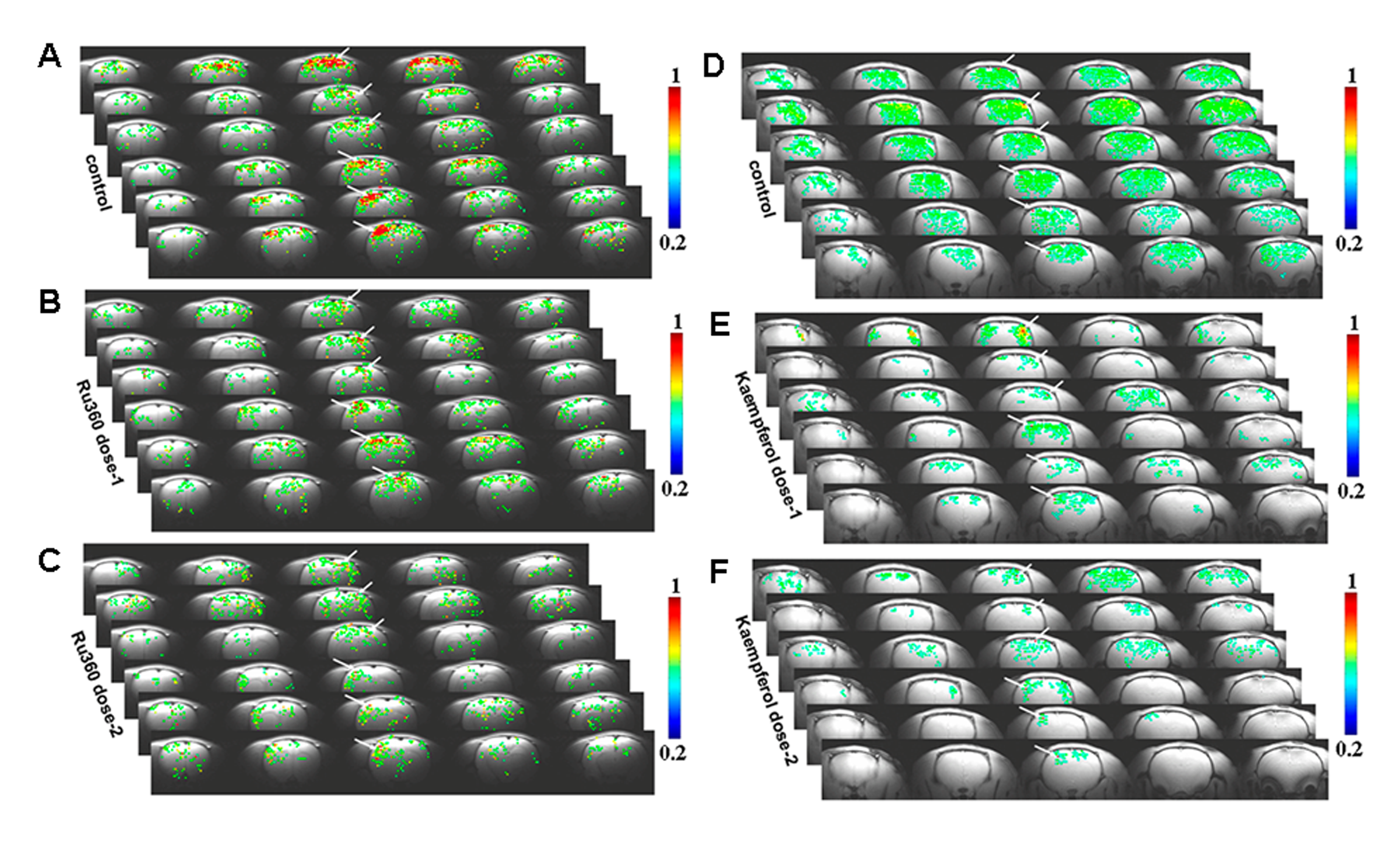

Supplement: Figure S1 — Typical resting state functional connectivity (RSFC) maps during mCU modulation. Seed voxels within the somatosensory cortex region of interest on either hemisphere were chosen at random and cross correlated with all voxels within the brain to generate six RSFC maps for each experimental condition. A–C. show the representative maps (correlation coefficient threshold of 0.2 corresponding to a P<0.0001; corrected for multiple comparisons) for control and Ru360 treated conditions (Ru360 dose-1 = 120 µg/kg; dose-2 = 240 µg/kg), E–F. show the same for control and Kaempferol treated (Kaempferol dose-1 = 1 mg/kg; dose-2 = 2 mg/kg). The seed voxel shows the highest correlation coefficient value in the maps (indicated by arrows). Six such correlation coefficient maps were averaged to determine the mean RSFC map for each experimental condition in an animal. A similar procedure was repeated for all animals. (TIF) [file pone.0063317.s001.tif]

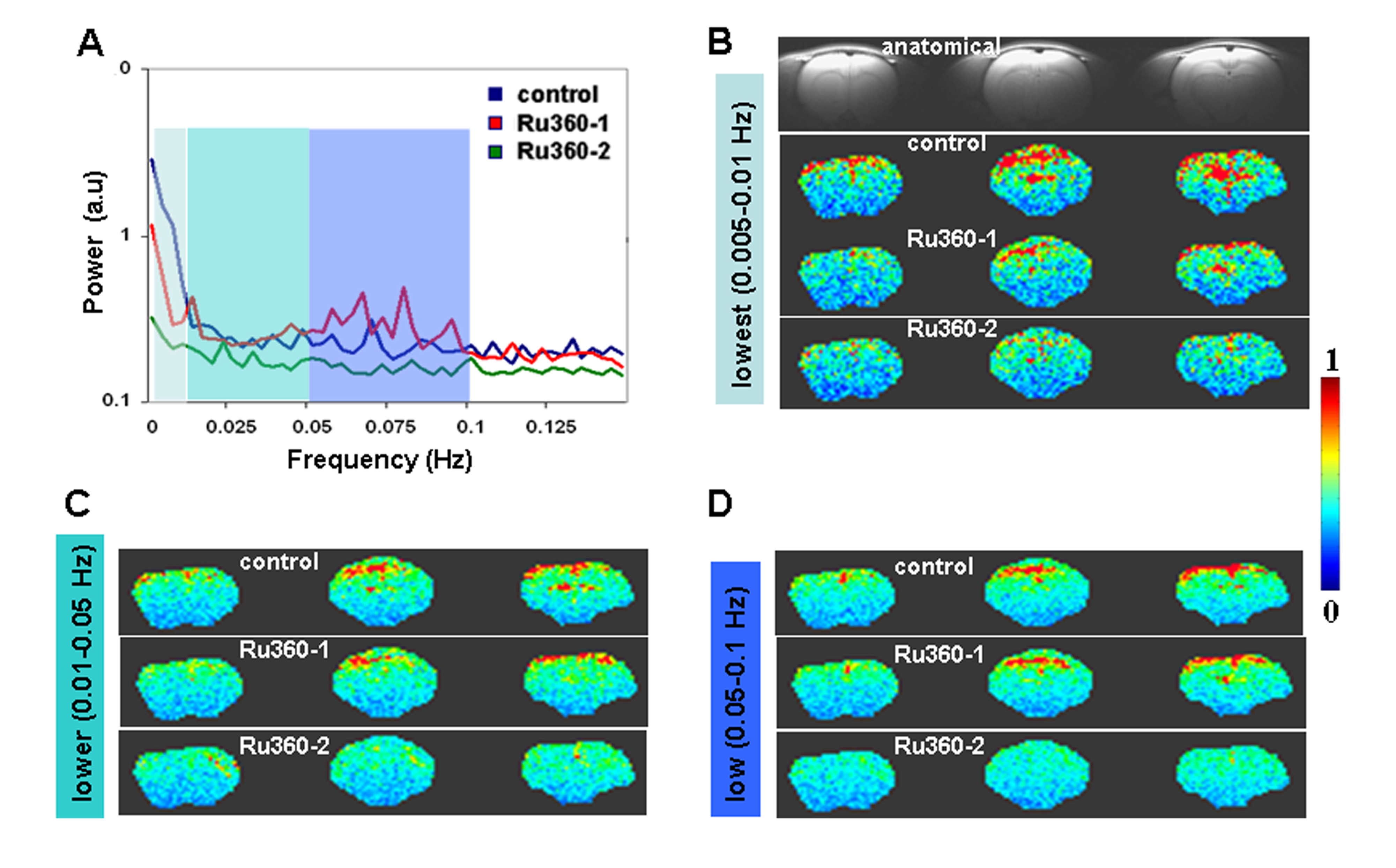

Supplement: Figure S2 — mCU modulation reduced the spectral power of the low frequency (<0.1 Hz) R-fMRI BOLD fluctuations. Inhibition of mCU activity with Ru360 (dose-1 = 120 µg/kg; dose-2 = 240 µg/kg) reduced the A. average power spectra (mean across voxels from the whole brain) in typical animals. R-fMRI spectral power were analyzed in three low frequency bands namely, lowest (0.005–0.01 Hz), lower (0.01–0.05 Hz) and low (0.05–0.1 Hz) indicated by different shades of blue color in panel A. B. the lowest band spectral power decreased during control and treatment with 120 µg/Kg and 240 µg/Kg Ru360 respectively. No significant change in the spectral power was observed between control and Ru360 treated states in the C. lower and D. low bands. (TIF) [file pone.0063317.s002.tif]

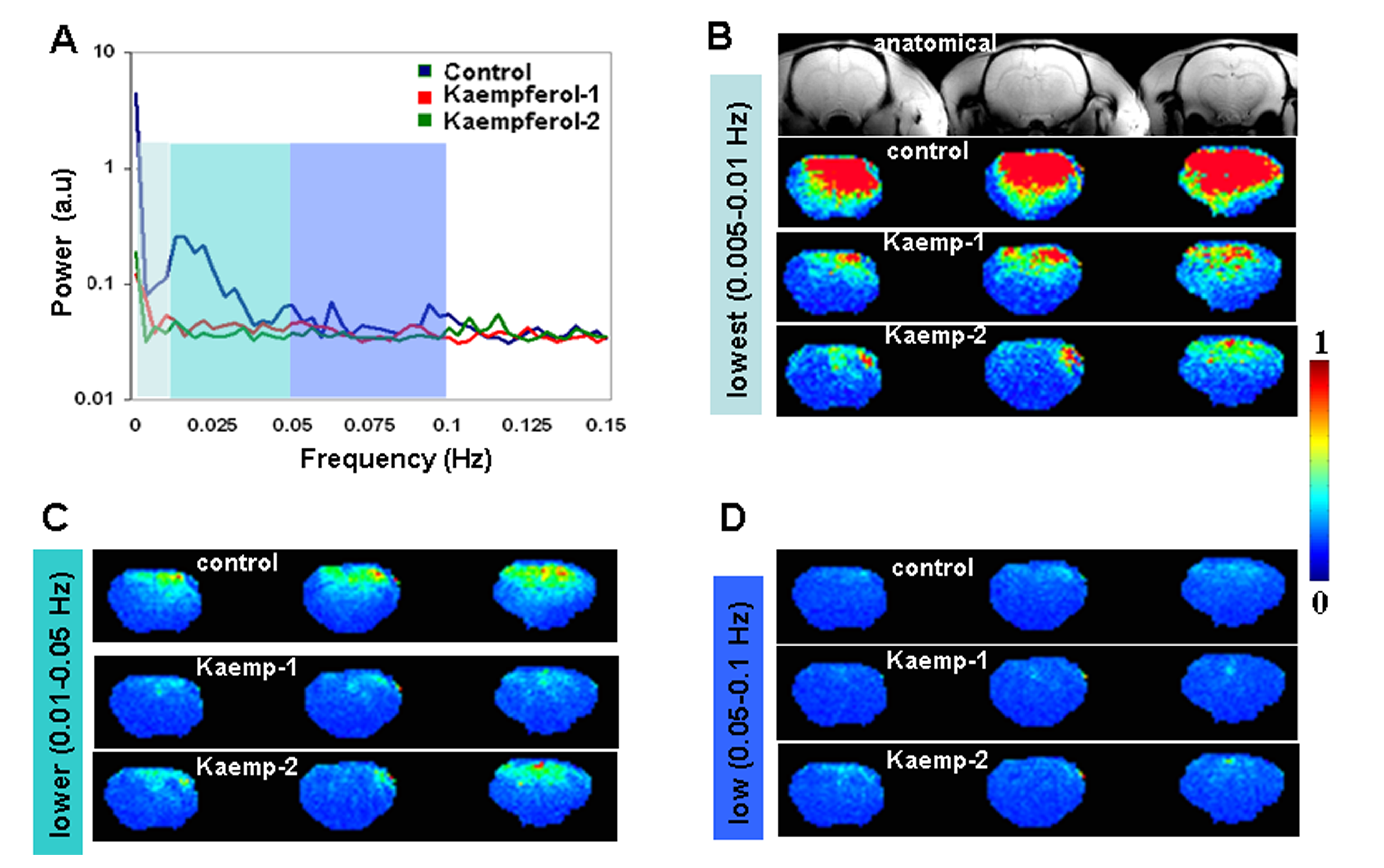

Supplement: Figure S3 — mCU modulation reduced the spectral power of the low frequency (<0.1 Hz) R-fMRI BOLD fluctuations. Enhancement of mCU activity with Kaempferol 1 mg/kg and 240 mg/kg Kaempferol reduced the A. average power spectra (mean across voxels from the whole brain) in typical animals. R-fMRI spectral power were analyzed in three low frequency bands namely, lowest (0.005–0.01 Hz), lower (0.01–0.05 Hz) and low (0.05–0.1 Hz) indicated by different shades of blue color in panel A. B. the lowest band and C. lower band spectral power decreased during treatment with 1 mg/kg and 2 mg/kg Kaempferol respectively with no effect on the D. low band spectral power. (TIF) [file pone.0063317.s003.tif]
